# Predictors of falls in patients during the first year after total hip arthroplasty: A prospective cohort study

**DOI:** 10.1002/hsr2.184

**Published:** 2020-08-19

**Authors:** Kazunari Ninomiya, Naonobu Takahira, Takashi Ikeda, Koji Suzuki, Ryoji Sato, Kazuo Hirakawa

**Affiliations:** ^1^ Department of Rehabilitation Shonan Kamakura Joint Reconstruction Center Kamakura Kanagawa Japan; ^2^ Sensory and Motor Control Kitasato University Graduate School of Medical Scienses Sagamihara Kanagawa Japan; ^3^ Department of Rehabilitation School of Allied Health Sciences, Kitasato University Sagamihara Kanagawa Japan; ^4^ Department of Nursing and Rehabilitation Sciences Showa University Tokyo Japan; ^5^ Department of Orthopaedic Surgery Shonan Kamakura Joint Reconstruction Center, Kamakura Kanagawa Japan

**Keywords:** falls, joint replacement, muscle strength, risk factor

## Abstract

**Background and Purpose:**

Since falls after total hip arthroplasty (THA) cause severe complications such as dislocation and fractures around the femoral stem, it is important to investigate what factors predict of falls. Thus, investigating predictors of falls in patients waiting for THA would be valuable as it lead to more strategic interventions to prevent these problems. The purpose of this study was to evaluate the predictors of falls in patients during the first year after THA.

**Methods:**

This is a prospective cohort study. A total of 157 patients who underwent THA for unilateral hip osteoarthritis were analyzed. The incidence of falls during the first year after THA was monitored, and patients were classified into a “faller” and “non‐faller” group. The following factors were compared between the two groups: demographic data (age, sex, body mass index, leg length discrepancy, length of hospital stay, and history of falling), preoperative hip abductor muscle strength, functional performance (single leg stance and maximum walking speed), pain during walking, and physical activity.

**Results:**

On multivariate logistic regression analysis, preoperative hip abductor muscle strength on the affected side and a history of falling were predictors of falls during the first year after THA. On subsequent receiver operating characteristic curve analysis, preoperative hip abductor muscle strength on the affected side was retained as a significant predictor, with a cut‐off strength of 0.46 Nm/kg differentiating the faller and non‐faller groups with a specificity of 73.6%, specificity of 50.0%, and area under the curve of 70.2%.

**Conclusions:**

Finding from the present study suggested that clinicians should focus on low preoperative hip abductor muscle strength on the affected side and a history of falling to prevent falls during the first year after THA.

## 1 INTRODUCTION

Total hip arthroplasty (THA) is an effective procedure for patients with end‐stage hip osteoarthritis (OA); it provides pain relief and improves muscle strength, functional performance and quality of life.^1^, ^2^ However, several years after surgery, lower extremity muscle strength and functional performance of patients after THA remain significantly less than that of healthy older adults.^3^, ^4^ In particular, weakness of hip abductor muscle strength causes instability during walking and abnormal gait patterns,^5^ which leads to a risk factor for falls.^6^, ^7^ Although falls occur in 15% to 24% of healthy older adults every year,^8^, ^9^ the incidence rate among patients after THA was specifically higher, ranging between 30% and 32%.^4^, ^10^, ^11^

Falls after THA may lead to a serious complications, such as periprosthetic fracture.^12^, ^13^ Reeves et al^14^ demonstrated that periprosthetic fracture was a difficult to treat and has high rates of hospital readmission, increased risk of mortality, and implying a significant burden to the healthcare cost. Thus, it is necessary to investigate factors associated with falls in THA patients to prevent falls.

One study showed that incidence and circumstances of these falls have been reported among patients before and after THA.^10^ Additionally, preoperative risk factors of falls in patients after THA were related to history of falls, depressive symptoms, reduced general health, and planned physical activity.^15^, ^16^ To our knowledge, little is known about the preoperative risk factors of falls, including lower extremity muscle strength and functional performance, after THA. Thus, the predictors of falling in patients undergoing THA will be valuable as it lead to more strategic physical therapy interventions to prevent falls. We hypothesized that preoperative hip abductor muscle strength on the affected side would be predictive falls during the first year after THA. The purpose of this study was to evaluate the predictors of falls in patients during the first year after THA.

## 2 METHODS

### 2.1 Study design

This is a prospective cohort study. We followed the strengthening the reporting of observational studies in epidemiology statement for reporting a cohort study.

### 2.2 Patients

Patients were recruited at our institute, between May 1 and December 24, 2016. The inclusion criteria were as follows: (a) scheduled primary THA for unilateral hip osteoarthritis; (b) scheduled for discharge to home within 5 days post‐surgery; (c) 45 to 75 years old; (d) no serious medical or orthopedic disease; and (e) no presence of dementia/psychological disorder. The exclusion criteria were as follows: (a) revision surgery; (b) scheduled for osteotomy or hip adductor muscle dehiscence, combined with THA; (c) complications during or after surgery; and (d) presence of a severe leg length discrepancy (>3 cm) before surgery.

All patients were treated with a primary THA using an antero‐lateral approach under general anesthesia combined with epidural anesthesia. A standardized multimodal protocol was used for postoperative analgesia. Intravenous acetaminophen (1000 mg) was first administered at the time of wound closure and then every 6 hours during the first 24 hours after surgery. Oral acetaminophen (1000 mg, three times a day) was then started. Celecoxib (400 mg) was given during the morning on post‐operative day 1; then, 200 mg was administered twice a day.

Postoperative rehabilitation was performed using a clinical path and booklet. Full weight‐bearing was allowed from the day of the surgery, with use of a walker initially, transitioning to the use of a cane on postoperative day 1 or 2. All patients were able to walk independently with a cane, and were discharged home within 5 days of the surgery. After discharge, they underwent regular follow‐up examination at 2, 6 months, and 1 year after surgery. At the time of the examinations, they were performed exercise instruction by a physical therapist for about 20 to 40 minutes. These exercises were performed open kinetic chain exercises (hip extension, external rotations, and abduction) aimed at improving the range of hip motion, increasing around these hip muscle strength. At the end of each intervention, the physical therapists instructed them to continue appropriate exercise at home. A specific rehabilitation program to prevent falls was not provided.

### 2.3 Ethical considerations

This study was approved by the institutional review board of the authors' affiliated institutions (TGE 00996‐115). We obtained verbal and written informed consent from all participants before enrollment. This study was conducted in accordance with the Helsinki Declaration.

### 2.4 Outcome measures

Demographic data of patients (age, sex, body mass index [BMI], leg length discrepancy, length of hospital stay, and history of falling) were recorded using a preliminary form. Three physiotherapists were separately responsible for each patient. Hip abductor muscle strength, functional performance (one leg stance time and maximum walking speed), hip pain during walking, and physical activity were evaluated at 1 month before surgery. Additionally, we assessed the incidence, circumstances, injury duration, and frequency of falls, prospectively, during the first year after surgery.

#### 2.4.1 Fall assessment

All patients completed a questionnaire recording the incidence of falls. Falls were defined as “a person falling onto the same level or a lower level on their own, without external force from other person, loss of consciousness, paralysis from a sudden stroke, or an epileptic seizure.”^17^ Additionally, the circumstances of the falls was also recorded, including the location (indoors, outdoors, or on stairs), time of day (morning, daytime, or nighttime), cause (tripping, slipping, or loss of balance), injury sustained (none, wound or bruise, or fracture), post‐operative duration (less than 6 months, or ≧6 months), and frequency of falls (1 time or ≧2 times) during the first year after surgery.^10^

#### 2.4.2 Hip abductor muscle strength

Hip abductor muscle strength was evaluated using a hand‐held dynamometer (MicroFET2, Hoggan Health Industries, Salt Lake City), with the patient in a supine position. The dynamometer was placed lateral to the fibula (2.5 cm proximal to the malleolus). Three trials of maximum effort were performed, and the highest value was used for the analysis. The torque‐to‐body weight ratio of abductor muscle strength (Nm/kg) was calculated from the body weight and spina‐malleolar distance.^18^ Good interrater and test‐retest reliability of handheld dynamometer measurements have been verified in previous studies in healthy adults^19^ and patients after THA.^20^

#### 2.4.3 One leg stance time

One leg stance time was measured in upright standing, with both hands held on the hips, starting at the time when one foot was lifted from the floor until one of the following criteria was met: (a) shifting in the position of the supporting foot on the floor; (b) the lifted foot touched the floor; (c) the lifted foot contacted the supporting leg; or (d) the maximum 30 seconds was reached. Measurements were conducted twice on both side, with the longer time used in the analysis.^21^

#### 2.4.4 Maximal walking speed

Maximal walking speed was measured along a straight distance of 10 m, with a 2‐m runway at both ends. Patients were instructed to “Walk as fast as you can.” Measurements were conducted twice, and the faster time was used in the analysis.^22^, ^23^

#### 2.4.5 Hip pain during walking

Hip pain during walking was evaluated using a 100‐mm visual analog scale score.^24^

#### 2.4.6 Physical activity

Patients were asked the mean number of days and hours of physical activity they performed in 1 week, using the International Physical Activity Questionnaire.^25^ The intensity of physical activity was classified as follows: high‐intensity exercise, 8 metabolic equivalents (Mets); moderate intensity, 4 Mets; and walking, quantified as 3.3 Mets. The activity intensity was converted to a respiratory quotient, and the number of calories consumed in activity per week was calculated from the respiratory quotient and body weight. In addition, based on the report by Brach et al,^26^ patients were classified into a high or low physical activity group (high, burning ≥1000 kcal, or low, burning <1000 kcal per week).

### 2.5 Sample size calculation

Based on a priori power analysis, the minimal sample size for the multivariate logistic regression analysis to examine significant factors (*α* = .05, power = .95, effect size = 0.3, potential predictor variables = 3) was calculated, assuming a 25% to 30% fall rate after THA. Hence, a sample of approximately 150 participants was required. To account for potential drop out (10%‐15%) over the 12‐month follow‐up period, a sample of 170 participants was needed.

### 2.6 Statistical analyses

The incidence rate of falling and fall‐related injuries was calculated. Initially, the following variables assessed before surgery were analyzed and compared between fallers and non‐fallers using Student's *t* tests or chi‐squared (*χ* ^2^) tests, as appropriate. Then, we performed a multivariate logistic regression analysis to assess the preoperative factors predicting falls during the first year after THA. For significant preoperative factors identified, a receiver operating characteristic (ROC) curve was constructed to determine their accuracy in distinguishing between fallers and non‐fallers, where accuracy was evaluated by the area under the curve, and the cut‐off value determined by the highest sum of sensitivity and specificity was used.

All statistical analyses were performed using SPSS Version 24 (SPSS, IBM, Inc, Chicago), and a *P* value < .05 was considered significant.

## 3 RESULTS

**Figure 1:**
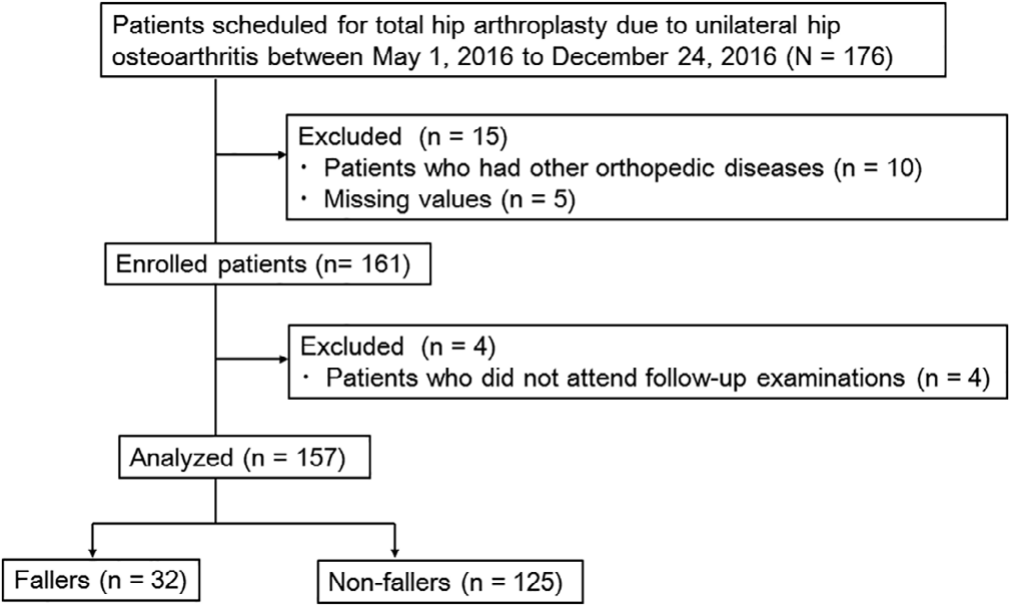
Flowchart of patients throughout the study

Patient selection and the flow of the study are shown in Figure 1. After excluding four patients who did not return for regular follow‐up examination, 157 patients (15 men and 142 women, mean age 63.9 ± 9.9 years) were included in analyses (follow‐up rate, 97.5%). None of these patients developed a postoperative infection or dislocation, and none required revision arthroplasty.

Comparison of demographic characteristics, hip abductor muscle strength, functional outcomes, and physical activity among fallers and non‐fallers are reported in Tables 1 and 2. The incidence of at least one fall occurred in 32 patients (20.4%). There was no significant difference in age, sex, BMI, leg length discrepancy, or length of hospital stay, between the two groups. Compared to non‐fallers, however, fallers had lower hip abductor muscle strength on the affected side (0.45 ± 0.17 vs 0.60 ± 0.22 Nm/kg, respectively; *P* < .001) and on the unaffected side (0.64 ± 0.22 vs 0.77 ± 0.26 Nm/kg, respectively; *P* = .008), and a more prevalent prior history of falling, with 37.5% of patients in the fallers group reporting a prior fall, compared to 17.6% in the non‐fallers group (*P* = .017).

**Table 1:** Patients demographic for post‐operative fallers and non‐fallers

	Fallers (n = 32) Mean ± SD	Non‐fallers (n = 125) Mean ± SD	*P* value
Age, years	64.0 ± 9.7	64.3 ± 10.0	.906
Female, %	96.9	92.8	.145
BMI, kg/m^2^	22.8 ± 3.3	23.4 ± 3.6	.349
Leg length discrepancy, cm	1.1 ± 0.9	1.1 ± 0.8	.692
Length of stay, days	4.9 ± 0.7	4.8 ± 0.7	.480
Falls history	37.5	17.6	.017
One or more falls in the 12 months prior to surgery, %			

Abbreviations: BMI, body mass index; SD, standard deviation.

**Table 2:** Comparison of muscle strength, functional performance, and physical activity before surgery between post‐operative fallers and non‐fallers

	Fallers (n = 32) Mean ± SD	Non‐fallers (n = 125) Mean ± SD	*P* value
Hip abductor muscle strength (Nm/kg)			
Affected side	0.45 ± 0.17	0.60 ± 0.22	<.001
Unaffected side	0.64 ± 0.22	0.77 ± 0.26	.008
One leg stance time (s)			
Affected side	14.48 ± 12.24	13.06 ± 12.40	.562
Unaffected side	20.18 ± 11.42	21.67 ± 11.20	.502
Maximal walking speed (m/s)	1.19 ± 0.37	1.21 ± 0.34	.803
Pain during walking (VAS) (mm)	22.38 ± 25.57	25.76 ± 26.19	.513
IPAQ			
High PA group^a^: Low PA group^b^ (n)	7:25	28:97	.579

Abbreviations: IPAQ, International Physical Activity Questionnaire; SD, standard deviation; VAS, visual analogue scale.

a High PA group: ≥1000 kcal/week.

b Low PA group: <1000 kcal/week.

**Figure 2:**
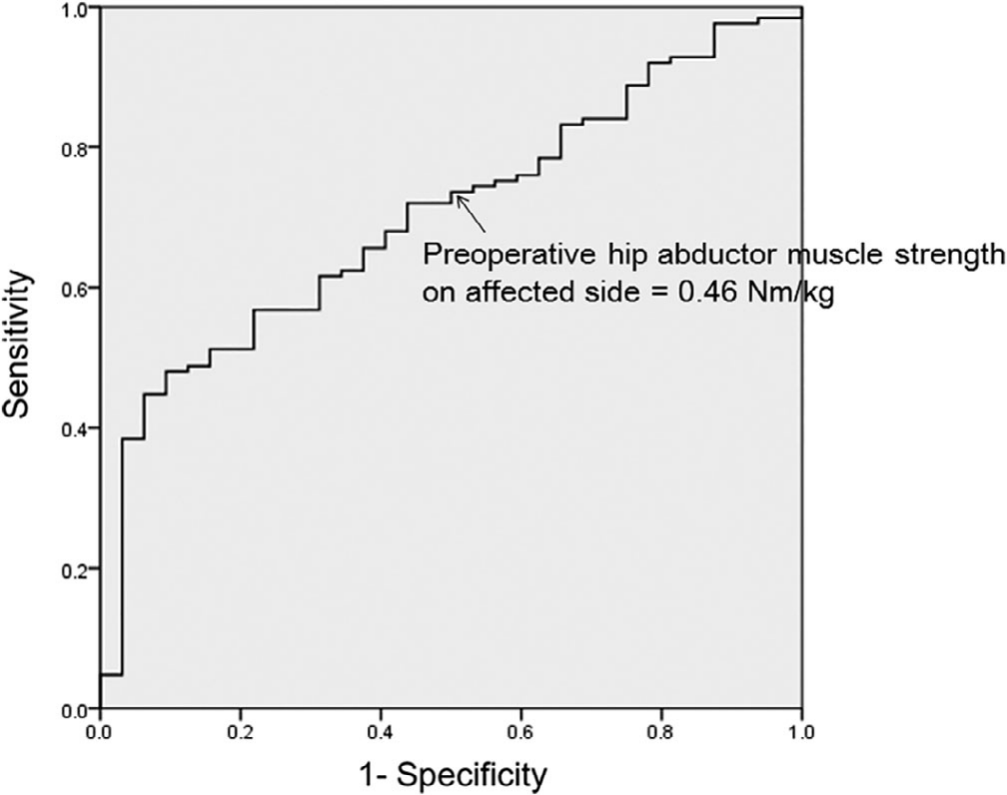
The area under the curve of 70.2%, with the optimal cut‐off value of the preoperative hip abductor muscle strength on affected side of 0.46 Nm/kg (sensitivity = 73.6%, specificity = 50.0%)

The results of the multivariate logistic regression analysis are summarized in Table 3. Preoperative hip abductor muscle strength on the affected side and a prior history of falling were independent predictors of a fall during the first year after THA. Figure 2 shows the ROC curves constructed to determine the optimal cut‐off value for preoperative hip abductor muscle strength on the affected side that *best* predicted a fall during the first year after THA. The cut‐off point of 0.46 Nm/kg for preoperative hip abductor muscle strength on the affected side yielded a moderate sensitivity and moderate specificity of 73.6% and 50.0%, respectively.

**Table 3:** Result of the multivariate logistic analysis (preoperative factors predicting falls during first year after THA)

	Odds ratio	95% confidence interval	*P* value
Hip abductor muscle strength on the affected side	1.04	1.003‐1.068	.031
Hip abductor muscle strength on the unaffected side	1.00	0.977‐1.027	.895
Falls history	.395	0.163‐0.958	.040

The circumstances of the falls after THA are reported in Table 4. Most first falls occurred indoors (46.9%), during the day (62.5%), and were due to tripping (65.6%). In 15 patients (46.9%), falls resulted in injuries, including a fracture in 2 patients (6.3%), namely a Colles' fracture in one and a periprosthetic femoral fracture in the other. Overall, 20 patients (62.5%) sustained an injury within 6 months of their THA, with two or more falls during the first year after THA reported by six patients (18.7%).

**Table 4:** Circumstances of the falls (only first fall reported if multiple falls)

Location	n (%)
Indoors	15 (46.9)
Outdoors	12 (37.5)
Stairs	5 (15.6)
Time	
Morning	4 (12.5)
Daytime	20 (62.5)
Nighttime	8 (25.0)
Cause	
Tripping	21 (65.6)
Slipping	6 (18.8)
Loss of balance	5 (15.6)
Injury	
None	17 (53.1)
Wound or bruise	13 (40.6)
Fracture (Colles' (n = 1) or periprosthetic femoral (n = 1))	2 (6.3)
Post‐operative duration	
less than 6 months	20 (62.5)
≧6 months	12 (37.5)
Frequency of falls	
1	26 (81.3)
≧2	6 (18.7)

## 4 DISCUSSION

The present study found that the incidence of falls was 20.4%, and the preoperative hip abductor muscle strength on the affected side <0.46 Nm/kg was significantly associated with falls during the first year after THA. To our knowledge, this is the first report of a specific factor of preoperative lower limb function predicting the risk of falling among post‐THA patients.

Several investigators have identified hip abductor muscle strength as a contributing factor to stability during standing and walking.^27^, ^28^ Ikeda et al^18^ reported that preoperative hip abductor muscle strength is strongly associated with hip abductor muscle strength after THA. Therefore, preoperative weakness of hip abductor muscles may also contribute to abnormal gait patterns, reduced stability during walking, and falls after THA. Moreover, in our present study, we identified that a preoperative abductor muscle strength <0.46 Nm/kg was associated with 70.2% probability of falling, which is a clinically important finding. Based on our result, we propose that a predictive cut‐off value of 0.46 Nm/kg has key implications for clinical outcomes of THA. Further research should be needed to clarify training methods that would be effective to improve preoperative hip abductor muscle strength in this clinical population.

Previous studies reported the prevalence of falls during the first year after THA was 25% to 32%^10^, ^11^, ^29^, which was similar to the results of the present study. Additionally, our findings that falls commonly occurred indoors, during the daytime, and resulted from tripping are consistent with the findings of Ikutomo et al^10^ who examined the incidence of falls in patients after THA (50.0% indoors, 66.2% during the daytime, 47.1% because of tripping, and 5.9% had fractures). Thus, our results were similar to those from previous studies among patients after THA.

History of falling was a predictor for postoperative falling in patients after THA,^15^, ^16^ which is consistent with previous knowledge. Additionally, Nagai et al^30^ demonstrated that a preoperative history of falling and fear of falling were related to walking ability after THA. This underlines the potential for a preoperative history of falling to induce a debilitating downward spiral, marked by loss of confidence, prolonged functional recovery, and an increased risk of falling after THA. It is important to further note that a preoperative history of falling is not only a risk factor for falling per se after THA, but also a factor of functional recovery after THA.

Finding from the present study suggested that surgeons and physical therapists should focus on low preoperative hip abductor muscle strength on the affected side and a history of falling to identify the most likely and important factors associated with increased fall risk during the first year after THA.

The limitations of our study need to be acknowledged. First, this was a single‐center study. Second, although preoperative hip abductor muscle strength is a fall‐risk predictor after THA, the relationship between other muscles and falls was not been examined. Third, the main cause of the fall was tripping, but we could not investigate the foot (affected or unaffected side) that was tripped. Fourth, we used an observational design which does not offer an intervention method for patients before surgery to improve hip abductor muscle strength. However, our findings do suggest that preoperative hip abductor muscle strength on the affected side and a preoperative history of fall might be useful indicators for preoperative screening of the risk for falls among patients after THA. Moreover, our findings to indicate the potential importance of implementing interventions to improve preoperative hip abductor muscle strength on the affected side as being necessary for preventing falls during the first year after THA. Further research is needed to increase other facilities and outcomes for preventing falls in patients after THA.

## 5 CONCLUSIONS

Our findings suggest that preoperative hip abductor muscle strength on the affected side was a fall‐risk predictor during the first year after THA. Therefore, surgeons and physiotherapists should be aware that patients waiting for THA with low hip abductor strength on the affected side and a history of falls were higher risk of falling and should provide interventions and advice for them to prevent falls.

## CONFLICT OF INTEREST

The authors declare no potential conflict of interest.

## AUTHOR CONTRIBUTIONS

Conceptualization: Kazunari Ninomiya, Takashi Ikeda

Data Curation: Kazunari Ninomiya, Takashi Ikeda

Formal Analysis: Kazunari Ninomiya, Takashi Ikeda

Investigation: Kazunari Ninomiya, Koji Suzuki, Ryoji Sato

Methodology: Kazunari Ninomiya, Takashi Ikeda, Koji Suzuki, Ryoji Sato

Writing‐Original Draft Preparation: Kazunari Ninomiya

Writing‐Review and Editing: Naonobu Takahira, Takashi Ikeda, Kazuo Hirakawa

Funding acquisition: Takashi Ikeda

All authors have read and approved the final version of the manuscript

Kazunari Ninomiya had full access to all of the data in this study and takes complete responsibility for the integrity of the data and the accuracy of the data analysis

## TRANSPARENCY STATEMENT

Kazunari Ninomiya affirms that this manuscript is an honest, accurate, and transparent account of the study being reported; that no important aspects of the study have been omitted; and that any discrepancies from the study as planned (and, if relevant, registered) have been explained.

## Data Availability

No original data are shared because of no informed consent for data sharing.
